# Formulation and Evaluation of Alginate-Based Hydrogel Membranes Loaded with Colistin for Effective Management of Multidrug-Resistant Wound Infections

**DOI:** 10.3390/pharmaceutics18010133

**Published:** 2026-01-21

**Authors:** Nizar Muhammad, Syed Sikandar Shah, Ashfaq Ahmad Shah Bukhari, Jamil Ahmed, Shahnaz Usman, Shujaat Ali Khan, Aftab Alam, Syed Arman Rabbani, Junaid Asghar

**Affiliations:** 1 Department of Pharmacy, COMSATS University Islamabad-Abbottabad Campus, Abbottabad 22020, Pakistan; nizarmuhammad432@gmail.com (N.M.); drshujatalikhan@cuiatd.edu.pk (S.A.K.); 2 Department of Clinical Pharmacy and Pharmacology, RAK College of Pharmacy, RAK Medical and Health Sciences University, Ras Al Khaimah 11172, United Arab Emirates; aftab@rakmhsu.ac.ae (A.A.); arman@rakmhsu.ac.ae (S.A.R.); 3 Department of Physiology, RAK College of Medical Sciences, RAK Medical and Health Sciences University, Ras Al Khaimah 11172, United Arab Emirates; ashfaq.ahmad@rakmhsu.ac.ae; 4 Department of Psychiatric Mental Health Nursing, RAK College of Nursing, RAK Medical and Health Sciences University, Ras Al Khaimah 11172, United Arab Emirates; jamil@rakmhsu.ac.ae; 5 Department of Pharmaceutics, College of Pharmacy, Salim Habib University, Karachi 74900, Pakistan; shahnazgauhar@gmail.com; 6 Faculty of Pharmacy, Near East University, Near East Boulevard, Mersin 10, Nicosia 99138, Turkey; muhammadjunaid.asghar@neu.edu.tr

**Keywords:** wound care biomaterials, sodium alginate hydrogels, antimicrobial wound dressing, multidrug-resistant infection management, clinical wound healing

## Abstract

**Background:** Combating antimicrobial resistance and developing dressings that match all aspects of wound healing will always be challenging. **Methods**: In this study, hydrogel membranes composed of sodium alginate (SA), polyvinyl alcohol (PVA), and Pluronic-f-127 (F-127) loaded with colistin (C) were formulated. The formulations were divided into two groups: group 1 (SA-PVA-C) and group 2 (SA-PVA-F127-C). **Results:** The membranes were characterized using multiple techniques, which confirmed component compatibility, physical cross-linking, an amorphous structure, and suitable surface morphology with acceptable porosity. Mechanical testing showed that both groups were suitable for wound-dressing applications. Differences in drug release across media (water, normal saline, and phosphate) were non-significant (*p* value > 0.05). Drug-loaded membranes (*n* = 3) from both groups showed antibacterial activity against multidrug-resistant Gram-negative *Pseudomonas aeruginosa* (ZOI = 20.33 ± 2.51 mm, 21.66 ± 2.08 mm). **Conclusions**: Overall, the developed hydrogel membranes (both group 1 and group 2) demonstrated promising in vitro potential as colistin delivery systems for wound infection management.

## 1. Introduction

The management of multidrug-resistant (MDR) wound infections is a concern worldwide because of poor prognosis and rising costs. In one study, 73% of patients had wounds with MDR infections, which significantly lengthened patients’ hospital stays [1]. A 2015 study found that the yearly expenditures of wound care were USD 50 billion in the United States, AUD 2.6 billion in Australia, and GBP 2.3 to 3.1 billion in the United Kingdom [2,3,4,5].

In a cross-sectional study of MDR infections, Gram-negative bacteria were 78.8% of isolates, while the remaining bacteria were Gram-positive. MDR infections are prevalent among Gram-negative bacteria in hospitalized burned patients, particularly *P. aeruginosa* [2]. In another study, *P. aeruginosa* caused 12/49 wound infections (17% MDR) [6]. According to another study, *P. aeruginosa*, Coagulase-negative *Staphylococcus*, and *Acinetobacter sp*. were the three most prevalent pathogens [7]. Colistin is commonly used against *P. aeruginosa* based on culture and sensitivity (C/S) reports of MDR wound infections [8].

Colistin (Polymyxin E), a lipopeptide antibiotic, is in the polymyxin class. It was discovered in 1947, and colistimethate sodium (a prodrug of colistin base activity, CBA) was approved for medical use in the US in 1970. Colistin is an option for the effective treatment of MDR Gram-negative infections due to its unique mechanism of action (Hydroxyl Radical Death Pathway, Vesicle–Vesicle Contact Pathway, and Membrane Lysis Death Pathway) [4]. A high incidence of adverse drug reactions (ADRs), particularly nephrotoxicity, has limited the use of colistin. The incidence of acute renal failure in 66 patients who received colistimethate sodium as an intravenous injection was 45% [9,10,11,12]. Colistin has been successfully administered to the lungs in aerosolized powder form to treat pneumonia in patients with cystic fibrosis [13,14]. Similarly, loading colistin into the chitosan-based hydrogel to treat infected wounds has also been studied [5]. Hydrogel loaded with antibiotics like vancomycin and polymyxin-B had previously been developed as a specialized wound dressing that kills existing bacteria, prevents their growth, and lessens the adverse effects of antibiotics while expediting tissue repair and regeneration [15].

Hydrogels are hydrophilic networks of polymers with three-dimensional forms and powerful mechanical characteristics. Since they include hydrophilic groups, they presumably expand when exposed to an aqueous solution. To heal a wound, hydrogel membranes function as a wet dressing, a paste component, and a debriding agent. Hydrogels donate moisture, due to which they produce more collagenase, raise the amount of moisture in necrotic wounds, and start the autolytic debridement process. Hydrogels used as drug delivery systems have evolved significantly from conventional formulations to modern systems that enhance therapeutic efficacy through controlled release mechanisms and improved biocompatibility [5,16,17].

Among the different natural polymers used in conventional and modern hydrogel formulations, sodium alginate (SA) has attracted particular attention because of its distinctive properties, which include safeguarding wounds from bacterial contamination, encouraging re-epithelialization, supplying a moist microenvironment for quicker wound closure, the formation of granulation tissues, biodegradability, biocompatibility, and the qualities of controlled release [18]. Other polymers, such as Pluronic F-127 (F-127), have unique properties such as temperature sensitivity and biodegradability, making them ideal for creating hydrogel-based wound dressings. Their unique thermoresponsive behavior enables easy application at lower temperatures and excellent gelation at body temperature, enabling ideal wound-healing settings [19].

This study aims to formulate two groups of alginate-based hydrogel membranes for the treatment of multidrug-resistant burn wound infections. Group 1 (SA-PVA-C) will consist of conventional alginate-based hydrogel membranes, while group 2 (SA-PVA-F127-C) will include modern thermoresponsive alginate and F-127-based hydrogel membranes. Both groups will be loaded with colistin as a treatment option for MDR wound infections. The study aimed to characterize the formulated hydrogel membranes using Fourier-transform infrared spectroscopy, X-ray diffraction, thermogravimetric analysis, mechanical strength testing, and scanning electron microscopy. In addition, thickness, solvent loss, water vapor transmission rate, swelling behavior, antibacterial activity, in vitro drug release, and gel fraction were evaluated to compare group 1 and group 2 formulations. We hypothesized that F-127 would enhance hydrophilicity, mechanical stability, and drug release behavior in group 2 compared with group 1.

## 2. Materials and Methods

### 2.1. Materials

#### Chemicals

Sodium alginate (SA; BDH Laboratory Supplies, Poole, UK), polyvinyl alcohol (PVA; average molecular weight ≈ 85,000–124,000 g/mol; 98–99% hydrolysis; BDH Laboratory Supplies, UK), Pluronic F-127 (Sigma-Aldrich, St. Louis, MO, USA), and colistin (Nabi Qasim Industries (Pvt.) Ltd., Karachi, Pakistan) were used. Glycerol, absolute ethanol, distilled water, sodium hydroxide (NaOH), potassium hydrogen phosphate (KH_2_PO_4_), hydrochloric acid (HCl), Mueller–Hinton agar, and sodium chloride (NaCl) were also used. All the chemicals were of analytical grade.

### 2.2. Methodology

#### 2.2.1. Method of Preparation of Alginate-Based Hydrogel Membranes

The solvent casting (physical method) with some modifications was used to formulate colistin-loaded alginate-based hydrogel membranes according to the content ratio given in Table 1. Briefly, for the preparation of prototype hydrogel membranes (F2, F9), sodium alginate (SA) 3% *w*/*w* and polyvinyl alcohol (PVA) 5% *w*/*w* were dissolved separately in distilled water at 50–75 °C while stirring continuously at 75–100 rpm until homogeneous solutions were obtained. F-127 (3% *w*/*w* ) was dissolved in cold distilled water (2–8 °C) until the formation of a clear solution. Colistin 250 mg/L was dissolved in distilled water [5,19].

For group 1, i.e., F1–F7 (SA-PVA-C), the colistin (C) solution was mixed with SA solution, and to the resultant SA-C solution, while constantly stirring, the PVA solution was added dropwise, and the resultant SA-PVA-C solution was stirred for 1 h at 75 rpm until a homogenized solution was formed whilst the glycerol (7% *w*/*w*) solution was added drop by drop into it.

For group 2, i.e., F8–F16 (SA-PVA-F127-C), the SA-C solution was added dropwise into the F-127 solution, stirring constantly without heating. Subsequently, to the resultant SA-F127-C solution, the PVA solution was added while stirring constantly till the formation of a homogenous SA-PVA-F127-C solution. After adding 7% *w*/*w* glycerol at the end, the mixture was sonicated to eliminate air bubbles created by mixing.

The solutions (30 g) were cast into Petri dishes (8.5 cm diameter) and dried overnight at 37 °C in the incubator. The membranes were then peeled from the Petri dishes and stored in airtight containers [19,20,21].

To clarify the repetition of certain formulations in Table 1, formulations F2 and F5 from group 1 and F9, F12, and F15 from group 2 are included to illustrate the incremental increase in the content ratio of each polymer, with F5 represented by F2, and both F12 and F15 represented by F9 throughout the analysis.

#### 2.2.2. Characterization

##### Fourier-Transform Infrared (FTIR) Spectroscopy

The FTIR spectroscopy method is used to assess the kind, structure, and formation of new bonds [22]. An FTIR Spectrophotometer (Cary630, Agilent Technologies, Santa Clara, CA, USA) was used for the FTIR spectroscopy. The polymers, monomers, and colistin were taken in powdered form, while the drug-loaded and blank hydrogel membranes were used in the form of thin films. A 100:1 solution of potassium bromide (KBr) was made with powdered polymers and colistin to examine powder samples, and it was then dried. A 65 kN pressure was applied for one minute after the mixture had dried completely at 45 °C to transform the powdered mixture into a semitransparent disk with a 12 mm diameter [23]. For analysis, spectrophotometers were mounted with a thin and fine film for the membrane samples [24]. The spectrum was captured at wavelengths between 4000 and 400 cm^−1^.

##### Thermogravimetric Analysis (TGA)

TGA is used for the thermal study of the polymer, monomer, blank, and colistin-loaded alginate-based hydrogel membrane. A Thermogravimetric Unit (STA8000, Perkin Elmer, Waltham, MA, USA) was used for TGA. The hydrogel composite was employed in its original state, while the powdered samples of SA, PVA, and F-127 were correctly ground into fine powder. The weight of the samples was maintained up to 10 mg, and they were then placed in a pan of platinum (100 µL) that was open and attached to a microbalance for accurate weighing. Dry nitrogen (20 mL/min) was used to purge the samples while the temperature was raised progressively (at a rate of 20 °C/min) from 30 to 800 °C [19,21,25]. TGA of colistin, SA, PVA, Pluronic F-127, and a few representative blank and drug-loaded hydrogel membranes (F7, F17, F2, and F9) were performed only because these samples offered enough information about the stability and thermal behavior of the drug, polymers, and formulated systems.

##### X-Ray Diffraction (XRD) Studies

The sample holder was filled with the powder drug sample, and the surface was leveled before placing the glass slide on top. The samples of empty membranes and hydrogel membranes loaded with drug were sliced in a way to cover the specimen holders, and the membrane thickness was approximately maintained at 0.5 mm. Colistin, polymers, blank, and drug-loaded formulations (F7, F17, F2, and F9) were all subjected to XRD examination. Since all the other formulations had comparable polymeric compositions, these formulations were selected as standard examples of each hydrogel group. As a result, further XRD examination of further samples was deemed unnecessary. The X-ray diffraction of the powder drug, unloaded, and loaded hydrogel membranes was performed through X-ray Diffractometer (JEOL JDX-3532 X-ray diffractometer (JEOL Ltd., Tokyo, Japan) [21,22].

##### Mechanical Strength

At room temperature, the produced hydrogel membranes’ tensile characteristics were assessed through a Universal Testing Machine (UTM) (100–500 KN, Testometric Inc., Rochdale, UK). For the investigation of the elongation break, elongation peak, elongation yield, and Young’s modulus, the membranes were sliced into dimensions of 17 to 18 mm width and 20 mm length. The binding strength was determined using a loading rate of 1 mm/min. The hydrogel membrane was securely connected to the extensometer and the load cell to accurately record the elongation and test load. The sample was subjected to tensile load stress until it was unable to exhibit the load and elongation curve. The findings reported are the average of three hydrogel membrane tests [19,24,26]. Equation (1) was used to calculate the percentage elongation [18].

(1) $$Elongation\;ratio\;\left(\%\right)=\frac{L}{L0}\times 100$$

In this case, “*L*” represents the pre-breakage maximum length, while “*L*0” represents the sample’s original length.

The following Equation (2) was used to compute *Young’s modulus* [18].

(2) $$Young’s\;modulus\;(E)=\frac{stress}{strain}$$

##### Scanning Electron Microscope (SEM) Analysis

Scanning electron microscopy was used to assess the surface morphology of the produced hydrogel membrane [27]. For this purpose, a scanning electron microscope (JSM5910, JEOL, Japan) was used. The membrane was adhered to the aluminum stub that was laced with gold using double-stick graphite tape with a typical thickness of ~300 Å. In order to record the surface morphology, accelerated current (10 kV) was used to scan the prepared sample at magnifications of 500, 1000, 5000, and 10,000 [28].

#### 2.2.3. In Vitro Evaluation

##### Thickness and Solvent Loss Analysis

The thickness of each hydrogel membrane was measured using a Vernier caliper at five different locations: four from the four edges and one from the middle.

To compute the *solvent loss* (% *w*/*w*), the following Equation (3) was used, for which the hydrogel membranes were weighed pre- and post-drying.

(3) % Solvent loss (g)=(Wi−Wf)/Wi×100

where *Wi* denotes the weight of the hydrogel’s final solution before casting or drying overnight at 37 °C, and *Wf* denotes the weight of the peeled hydrogel membranes when prepared [29].

##### Water Vapor Transmission Rate (WVTR)

WVTR represents the membrane’s ability to transport moisture. For this purpose, 10 mL of distilled water was added to 18 mm diameter vials. The entrance of the vials was covered with a circular patch of hydrogel membranes that were cut out of each batch. Following that, Teflon tape was used to precisely air-tighten these vials. To estimate the *WVTR*, Equation (4) was used, in which the vials containing distilled water were weighed before being kept in the incubator at 37 °C for 24 h and were weighed again after 24 h [5].

(4) $$WVTR=(Wi-Wf)/(A\times 24)\cdot 106\;(g/m^{2}\cdot 24\;h)$$

where “*Wi*” and “*Wf*” represent the beginning and ultimate weights of the water-containing vials, respectively, while *A* represents the area of the vial’s orifice on which the hydrogel film was applied.

##### Swelling Studies

Samples (2 × 2 cm area, dried for 24 h. in the incubator) of the prepared formulation were weighed before dipping into 20 mL each of the required media, i.e., phosphate buffer, normal saline, and distilled water, and were weighed again post dipping at predefined time intervals (0, 10, 20, 30, 40, 50, and 60 min) [20]. Minor modifications were made to the reported method because direct handling of the samples in the medium can be difficult and may result in sample loss. To support each sample, a filter paper (2.5 × 2.5 cm) was wetted in the medium and weighed. The wetted filter paper was placed in a dry Petri dish, and the sample was placed on top. The medium (20 mL) was then added carefully without disturbing the sample. At each time point, the sample and filter paper were removed and gently blotted to remove surface liquid. Sample weight was calculated by subtracting the filter paper weight from the combined weight. The degree of swelling (*q*) was calculated using Equation (5).

(5) $$Swelling\;Ratio\;(q)=\frac{Ws}{Wi}$$

where “*Wi*” is the original weight of the sample after drying in the incubator for 24 h and “*Ws*” is the weight of the sample after soaking for a specified time in the medium [20].

##### Percentage Gel Fraction (GF%)

The membranes were divided into pieces of 4 cm^2^, dried in an incubator for 6 h, and then weighed (*W*0). After being dried, samples of the hydrogel membrane were submerged in distilled water until swollen to equilibrium or no more weight gain was seen. The materials were weighed again after being dried in the incubator overnight at 37 °C (*We*). The following Equation (6) was used to calculate the percentage gel fraction (*GF*%) [19]:

(6) Gel fraction (GF%)=(We/W0) × 100

##### Determination of Drug Loading

The drug (colistin 250 mg/L) was loaded into the hydrogel membranes (both groups) using a pre-drug loading technique; therefore, no drug was lost in the process [5]. The final solution before casting into membranes was 30 g for one hydrogel membrane, which contained 7.5 mg (93,500 IU) of colistin. For drug release studies, the developed hydrogel membranes were round, having a surface area of 57 cm^2^ (radius 4.25 cm), out of which 2 × 2 cm (4 cm^2^) membrane from each formulation was used. Therefore, if 57 cm^2^ has 7.5 mg of colistin, then 4 cm^2^ will have approximately 0.52 mg (520 µg, 3125 IU) of colistin.

##### In Vitro Drug Release Analysis

An in vitro drug release analysis of colistin-loaded hydrogels was carried out. Dialysis bags (12,000–14,000 Daltons) were filled with colistin-loaded hydrogel membranes already cut into a 2 × 2 cm^2^ size. The dialysis bag was meticulously sealed to prevent any media from entering or exiting the bag directly. Water, normal saline (0.9% NaCl), and phosphate buffer (pH 7.4) media were used in the drug release investigation. The three different release media were used to simulate different physiological environments to predict the in vivo behavior of the developed hydrogel membranes regarding drug release. Although water does not closely mimic physiological conditions, it was used as a simplified medium to assess drug release without interference from ions or buffer components. Because the membranes are intended for wound application, normal saline (0.9% NaCl) was used to mimic the ionic strength and osmolarity of extracellular fluid at the wound site. Furthermore, the phosphate buffer (pH 7.4) was used to provide an approximation of the physiological pH of extracellular fluids (wound site) for predicting the in vivo drug release behavior.

Sealed dialysis bags were fully submerged in 25 mL of release medium to maintain sink conditions. The media containers were shaken on orbital shakers (50 rpm) at room temperature, and 5 mL of the sample was taken and replaced with fresh media at set time intervals (0.5, 1, 2, 3, 4, 6, 8, 12, 16, 20, 24, 30, and 36 h). The samples that were taken out were measured at 280 nm using a UV spectrophotometer. By comparing the outcomes to the colistin standard curve, the total drug release was computed. The following Equation (7) was used to determine how much colistin was released from the sample hydrogel membranes [19,30]:

(7) $$Drug\;release\;\%\;=\left(\frac{The\;amount\;of\;released\;drug}{The\;amount\;of\;loaded\;drug}\right)\;\times 100$$

##### Antibacterial Study

An antibacterial study was carried out through the disk diffusion method using Mueller–Hinton agar [5]. After being prepared and autoclaved, the agar medium was put into Petri dishes, in a laminar hood, to a thickness of 4 mm. Subsequently, antibiotic-resistant but colistin-sensitive *P. aeruginosa* strains, obtained from (Pathology Department, Saidu Group of Teaching Hospitals, Swat, Pakistan), were lawn-cultured on agar plates. Disks of the developed hydrogel membranes were prepared in the order of F7 (blank), F2 (drug loaded), F17 (blank), F9 (drug loaded), all having a diameter of 8 mm. The F2 and F9 formulations, as per the area of the disk, contained approximately 65 μg of colistin. A positive control of commercial colistin 5 mm diameter (10 μg colistin) disk and a sterilized blank filter paper disk as a negative control were also taken. The prepared disks were then placed 24 mm apart from each other on the plates accordingly. The procedure was performed in triplicate. After 24 h in an incubator at 37 °C, the zones of inhibition (ZOI) were measured in mm using a ruler [5,19].

*P. aeruginosa* was chosen as the test organism because of its well-established multidrug resistance profile and high frequency in burn wound infections. Furthermore, rather than examining the antimicrobial spectrum, the antibacterial assessment in this study was meant to verify the colistin’s continued efficacy following inclusion into the hydrogel matrix and to evaluate formulation performance.

#### 2.2.4. Statistical Analysis

All physicochemical, mechanical, and bactericidal tests were carried out in triplicate (*n* = 3), and all formulations were made using the same procedures. The results, which show adequate consistency and reproducibility of membrane production and performance, were reported as mean ± standard deviation [31]. Using OriginPro 2018, the Kruskal–Wallis test (a nonparametric analysis of variance) was applied to determine differences in percent drug release among formulations within each medium [32]. Descriptive statistics were applied to report the in vitro antimicrobial studies (*n* = 3).

## 3. Results and Discussion

### 3.1. Physical Appearance

**Figure 1:**
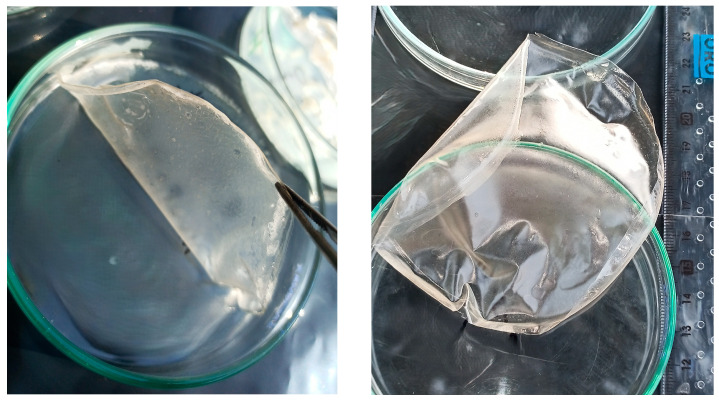
Physical appearance of the prepared hydrogel membranes (group 1).

**Figure 2:**
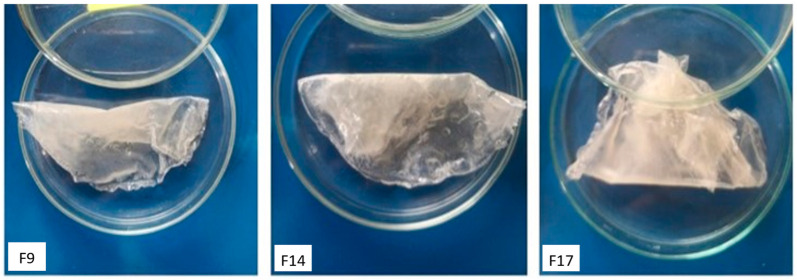
Physical appearance of the prepared hydrogel membranes (group 2).

The hydrogel membranes in group 1 (F1–F7) were elastic, shiny, uniformly thick, and transparent light brown (Figure 1). In contrast, the group 2 membranes (F8–F17) were less elastic, non-shiny, uniformly thick, and cloudy/turbid white (Figure 2). This difference was attributed to the presence of Pluronic F-127 in group 2. All the membranes remained stable during storage, with no noticeable change in color or texture.

### 3.2. Characterization

#### 3.2.1. Fourier-Transform Infrared (FTIR) Spectroscopy

**Figure 3:**
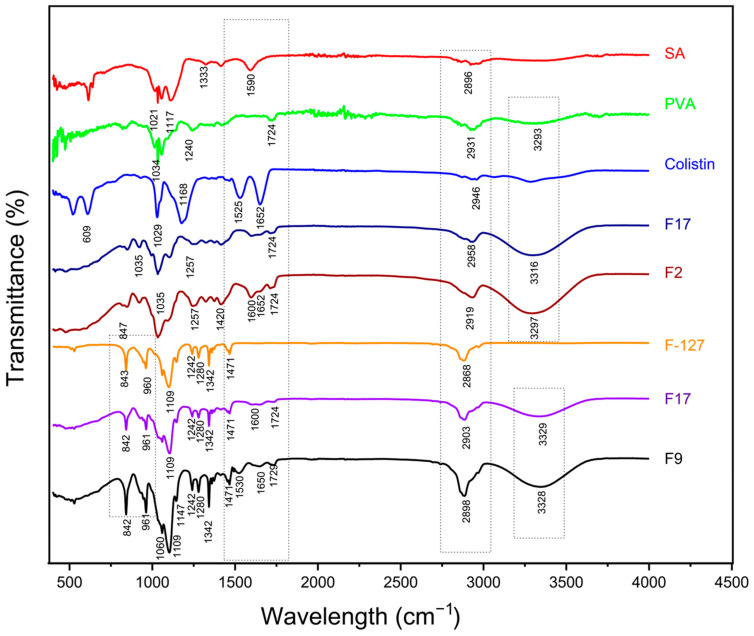
FTIR Spectra of SA, PVA, colistin, F7, F2, F-127, F17, and F9.

The FTIR spectrum (Figure 3) of SA showed peaks at 3259 cm^−1^, 2896 cm^−1^, 1590 cm^−1^, 1400 cm^−1^, and 1033 cm^−1^ which indicates presence of the -OH group, -CH aliphatic group, -COO (asymmetric), -COO (symmetric) of the carboxylic group (C=O), and the stretching vibration of -CO group, respectively [22,33]. The spectrum of PVA showed peaks at 3239 cm^−1^, 2931 cm^−1^, 1724 cm^−1^, 1240 cm^−1^, and 1035 cm^−1^, which indicates presence of the -OH group, -CH aliphatic group, stretching vibrations of the -CO group, wagging vibrations of -C-H as (CH-OH) coupling, and the -CO group, respectively [33]. The spectrum of colistin powder showed distinguishable characteristic peaks at 1652 cm^−1^, 1525 cm^−1^, 1168 cm^−1^, and 1029 cm^−1^, which are characteristic of the amide N-H bending and stretching vibrations of the C-N group [34]. A wideband at 2868 cm^−1^ was visible in the spectrum of F-127 and was attributed to the stretching vibrations of the -CH group. The CH2 and CH3 groups were linked to the 1342 cm^−1^ peak, while the peaks at 1109 cm^−1^ were connected to the polymer’s ether group. The peak shown by the alkenes group (=C-H) was at 960 cm^−1^ [20].

In comparison to its parent components, the blank hydrogel membranes, i.e., F7 and F17, displayed distinct spectra. Peaks at 3316 cm^−1^ and 2958 cm^−1^, 1597 cm^−1^, 1257 cm^−1^, 1020 cm^−1^, and 847 cm^−1^ proved the existence of -OH group, -CH group, stretching vibrations of -CO group, and wagging vibrations of -C-H in (-CH-OH) coupling in the structure, respectively, in F7’s spectrum [20]. However, some of the observed peaks in both F7 and F17 were slightly different from those of the parent compounds, but most of the peaks matched, which proved physical cross-linking of the hydrogel components. Distinctive peaks of colistin were observed in colistin-loaded hydrogel membrane’s spectra (F2 and F9), which confirmed that no chemical interaction occurred between colistin and the formulated hydrogel membranes. The findings support the hypothesis that the formulations can serve as effective carriers for colistin, ensuring its stability and bioavailability [35].

#### 3.2.2. Thermogravimetric Analysis (TGA)

**Figure 4:**
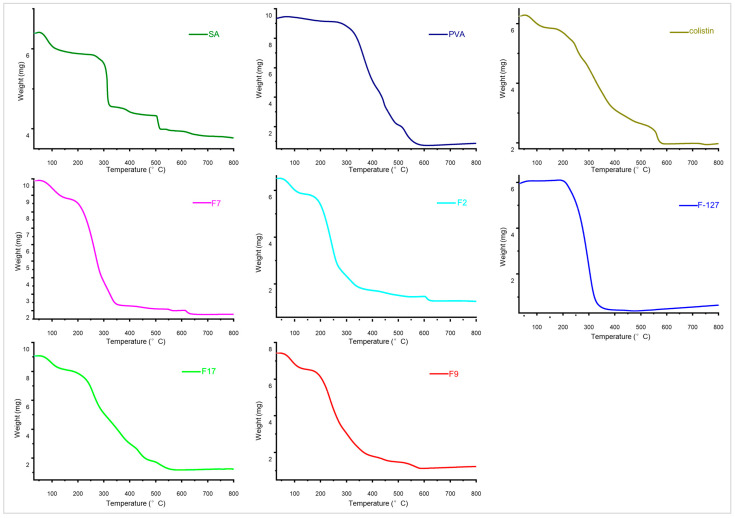
Thermogravimetric analysis (TGA) thermograms of SA, PVA, colistin, Pluronic F-127, and selected blank F7, F17, and drug-loaded hydrogel membranes F2, F9.

The thermogram of SA showed that it started losing weight at 50 °C and continued to lose weight until it reached 280 °C. During this temperature range, there was a total weight loss of 15%. This weight loss appears to be due to the release of stored water and the breakdown of mannuronic and glucuronic acids [25]. Subsequently, a 35% loss in weight was caused by the deterioration of the major backbone and organization of SA, which occurred between 280 °C and 300 °C as shown in Figure 4. SA breakdown began at 300 °C and progressed to 500 °C, with the total weight lost reaching 70% at this point. From 500 °C to 700 °C, a 10% mild breakdown occurred [33].

According to PVA’s thermogram, when the temperature increased from 60 °C to 320 °C, just 10% of the initial weight was lost due to the removal of water molecules from the structure. The side chains and main backbone of PVA broke during the second stage of weight loss, which occurred between 320 °C and 580 °C and led to a 75% weight loss. A further 10% mild disintegration took place between 580 °C and 630 °C [25]. The initial weight loss in PVA up to 320 °C is mainly due to the release of absorbed and bound water, but small structural changes cannot be completely ruled out. The main thermal degradation of the PVA backbone is observed at temperatures higher than 320 °C, in accordance with previous works [25].

The initial weight loss in F-127 was 5% from 60 °C to 230 °C, followed by 90% destruction at 325 °C, despite the fact that the breakdown process began at 235 °C. A further 10% disintegration took place between 325 °C and 400 °C [36]. The thermal degradation pattern observed in F2 and F7 formulations (group 1) showed that initially, 15% of the material degraded between 80 and 150 °C, likely due to the loss of water. Then, 75% of the degradation happened between 200 and 320 °C, which was caused by the breaking of bonds within and between molecules. The rest of the degradation occurred at temperatures ranging from 350 to 600 °C [25]. Thermograms of F9 and F17 formulations (group 2) showed an initial 15% degradation at 80–150 °C as stage one, 75% degradation at 200–500 °C due to degradation of the core molecules, and the remaining 10% at 500–600 °C [23,36]. In conclusion, the TGA results indicate that while both formulations (group 1 and group 2) exhibit thermal degradation characteristics, the inclusion of F-127 significantly alters their thermal behavior, potentially leading to improved performance of group 2 formulations due to better mechanical stability [19].

#### 3.2.3. X-Ray Diffraction (XRD) Studies

**Figure 5:**
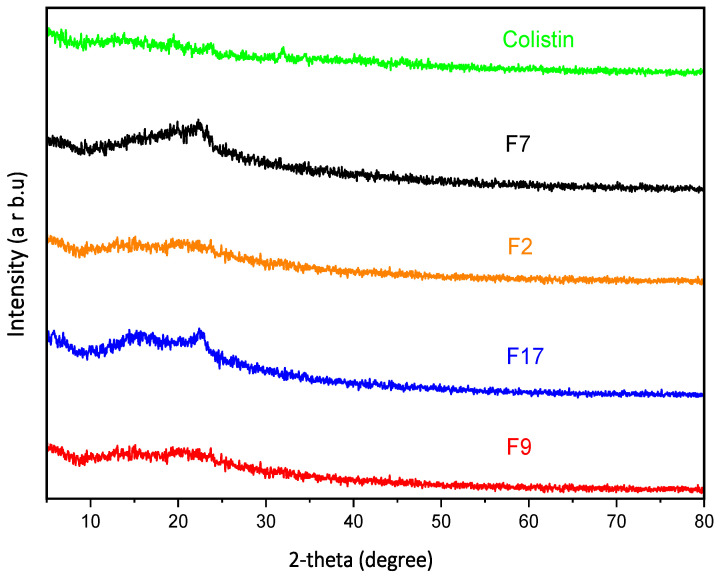
XRD diffractogram of colistin, F7, F2, F17, and F9.

The XRD diffractograms of colistin and the developed hydrogel membranes F7, F2, F17, and F9 were obtained as given in Figure 5. According to the XRD data, the given colistin sample appeared to be amorphous without any discernible crystalline peak across the scanning range [37]. No distinguishing peaks were seen throughout the developed hydrogel membranes, which indicated their amorphous nature. The diffraction pattern confirmed the drug’s compatibility with the hydrogel membrane. Furthermore, the sustained release mechanism anticipated from these amorphous formulations aligns with the objectives of developing effective antimicrobial treatments, particularly in localized applications where prolonged drug action is essential [21].

#### 3.2.4. Mechanical Strength Analysis

**Figure 6:**
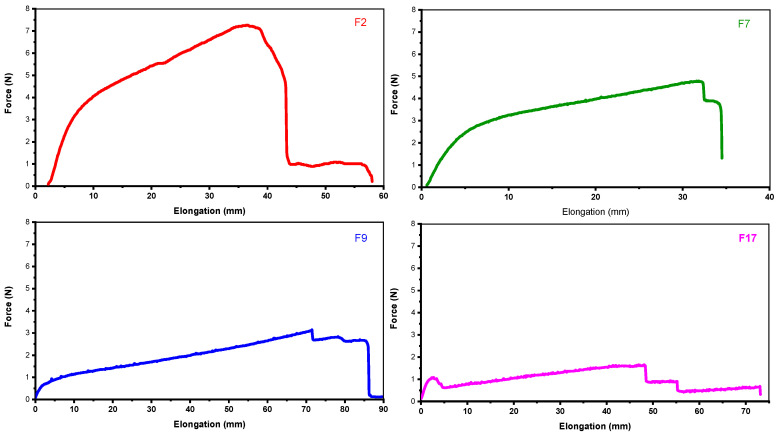
Graphical representation of the mechanical properties as per UTM results of F2, F7, F9, and F17.

The developed hydrogel membranes have to be applied to external wounds as a dressing and shall endure the external environmental conditions for which good mechanical qualities are essential [24]. Therefore, the mechanical properties of both the blank and drug-loaded hydrogel membranes were studied as shown in Figure 6. Details about percent elongation at break, tensile strength, peak load, and Young’s modulus are given in Table 2. Tensile strength and percentage elongation at break are used to calculate the maximum strain and stress that a membrane can sustain before breakage. Peak load is the point at which the membrane ruptures entirely. The Young’s modulus describes the amount of elasticity that a membrane can withstand [38]. Any wound dressing will exhibit good flexibility, ductility, and strength to the external environment if its tensile strength and percentage elongation are high, and its Young’s modulus is low.

Before mechanical testing, all hydrogel membranes were dried in an incubator at 37 °C to a constant weight. The residual water content of the tested samples was <5% (*w*/*w*), which guaranteed similar and reproducible mechanical measurements.

In group 1, the drug-loaded formulation F2 exhibits a significantly higher percentage elongation compared to the blank formulation F7; this may be due to the additional physical cross-linking between the polymers SA and PVA, caused by colistin. Higher elongation percentage indicates that F2 is more flexible and may be advantageous as a wound dressing. Furthermore, F2 also demonstrates a higher tensile strength and peak load compared to F7, potentially due to the presence of colistin and physical cross-linking, making it potentially more suitable for load-bearing applications. Interestingly, while F2 has a higher tensile strength, its Young’s modulus is comparatively lower than that of F7. This indicates that while F2 can withstand greater loads, it is less stiff than F7, suggesting that F2 may be more compliant [39,40,41].

In group 2, the tensile strength, peak load, and Young’s modulus of F9 are higher compared to F17, reinforcing the observation that the drug-loaded membrane, potentially due to additional cross-linking among the polymers, can handle more stress before yielding and that it is stiffer and less prone to deformation under load [41,42]. The presence of Pluronic-127 (F-127) in F9 and F17 could contribute to their mechanical properties through enhanced hydrophilicity and elasticity due to the block copolymer structure of F-127. Additionally, the presence of F-127 improves the flexibility and water retention of hydrogels, which might explain the relatively lower tensile strength, but higher elongation compared to F2 and F7, lacking the Pluronic-127, possibly due to a more rigid polymeric network formed by their respective compositions. This difference highlights how formulation choices significantly influence the mechanical characteristics of hydrogel membranes [43,44]. Moreover, in comparison to the Young’s modulus values of the prepared hydrogel membranes, the Young’s modulus of normal skin varies between 0.42 MPa and 0.85 MPa for torsion tests, and between 4.6 MPa and 20 MPa when extracting stress values were obtained in tests carried out using mechanical equipment [45]. Conclusively, it is demonstrated through the mechanical strength analysis that the developed hydrogel membranes have excellent mechanical wound-dressing properties, both in the form of flexibility (as seen in F2) and stiffness (as seen in F9) [31,46].

#### 3.2.5. Scanning Electron Microscope (SEM) Analysis

**Figure 7:**
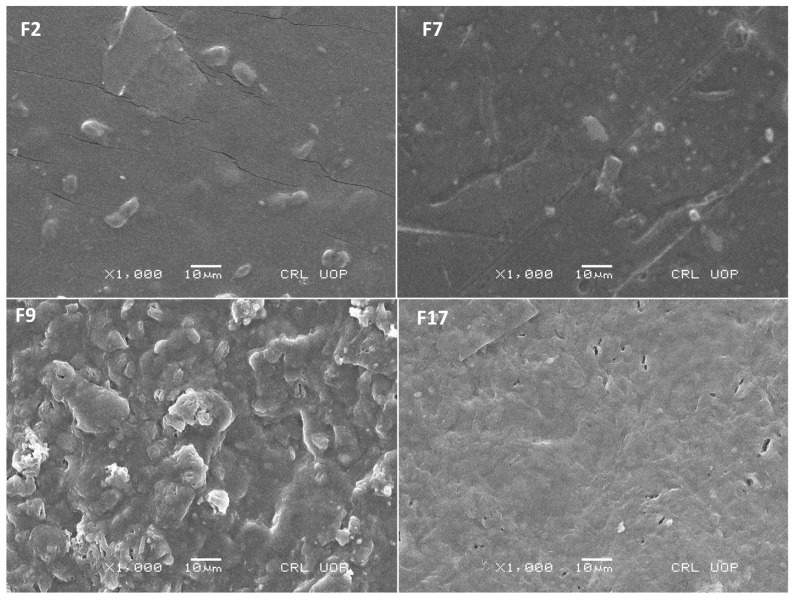
SEM findings at ×1000 resolution for the created hydrogel membranes F2, F7, F9, and F17.

Scanning electron microscopy is regarded as the most promising of the advanced topographic investigations because it provides crucial insights into the surface structure and other morphological aspects of the synthesized material [47]. SEM images obtained both at high and low resolutions for F7 and F2 showed smoothness and no porosity, clearly indicating that polymers have been successfully cross-linked (physically) and the drug is incorporated into the formulations (Figure 7). Conversely, the images obtained for F17 and F9 confirmed somewhat porous, loose, dense, and rather coarse surfaces. This structure demonstrated that the polymers had effectively undergone physical cross-linking, as evidenced by the hydrogels of SA and those of F-127 and PVA [48,49]. At high magnification, such as 5000× and 10,000×, there were tiny pores in the structure, considered to be advantageous as oxygen supply is not completely hindered by the membrane, and it would increase wound healing by ensuring that the wound receives an optimal supply of oxygen [50]. In conclusion, the addition of F-127 appears to be beneficial for the wound environment, potentially due to the tiny pores in group 2 formulations, which will not permit any bacteria to go through the membrane, and any type of infection may be prevented, assisting in the oozing of an ideal quantity of exudates and supply of oxygen for wound healing [24].

### 3.3. In Vitro Studies

#### 3.3.1. Thickness and Solvent Loss

The thickness and solvent loss of each of the developed hydrogel membranes from F1 to F17 were measured in triplicate; the results are given in Table 3, which has yielded significant insights into the relationship between polymer concentration and membrane characteristics. The surface on which the Petri dishes were set needs to be sufficiently leveled to prevent any type of fluctuation in thickness [21]. The highest average thickness (0.17 mm ± 0.03) was noted for F9, while the lowest thickness was recorded for F7 and F17. Moreover, an increase in thickness can also be correlated with a decrease in solvent loss because of an increase in the concentration of various polymers for the range of developed hydrogel membranes. The lowest percent solvent loss was observed in F9, followed by F2. The production of hydrogen bonds between the hydrophilic polymers, e.g., SA, F-127, and PVA, and solvent (water) increases with higher concentrations of polymers, as do the hydrophilic groups, or -OH groups, which results in reduced solvent loss and hence enhances the thickness of the hydrogel membranes [21]. The results indicate a clear trend: as the concentration of SA, PVA, and Pluronic F127 increased, both the thickness of the membranes and the reduction in solvent loss were observed.

#### 3.3.2. Water Vapor Transmission Rate (WVTR)

**Figure 8:**
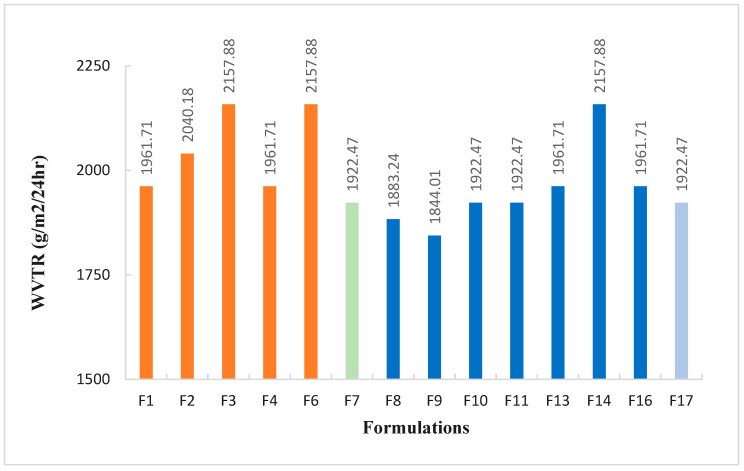
Water vapor transmission rate of the developed hydrogel membranes F1 to F17.

After recording the required weights of the vials as per methodology, WVTR for each developed hydrogel membrane was calculated, as expressed in Figure 8. The ability to retain moisture is reflected by the water vapor transmission rate [51]. An ideal wound dressing should possess properties that should maintain adequate oxygen and moisture transmission, prevent dehydration, and create the ideal environment for wound healing [20]. Human skin has a normal WVTR of 204 g/m^2^/24 h, but when skin is injured, the rate changes and can range from 279 g/m^2^/24 h to 5138 g/m^2^/24 h for minor burns and crushing wounds, respectively. Furthermore, the WVTR for ideal wound dressings ranges from 2000 to 2500 g/m^2^/24 h [2,21].

The formulations F3, F6, and F14 exhibited the highest WVTR at 2157.88 g/m^2^/24 h, closely followed by F2 at 2040.18 g/m^2^/24 h. All the other formulations, as shown in Figure 8, also exhibited permissible WVTR required for a wound dressing, where maintaining an optimal moisture level is essential for healing. The results of WVTR for membranes can be influenced by several factors, such as porosity, hydrophilic content, thickness, and composition [51,52,53]. The presence of hydrophilic groups within the formulation enhances water vapor permeability due to their ability to interact with water molecules, facilitating easier transport through the membranes [53]. The difference in WVTR observed among the produced formulations could also be related to the variable thickness of the membranes utilized in the analysis. Thinner membranes often exhibit higher WVTR values due to reduced resistance to vapor flow; therefore, despite a low comparative porosity of group 1 formulations, a converse trend of higher WVTR was observed in group 1 formulations compared to some of the group 2 formulations [51,52].

#### 3.3.3. Percentage Gel Fraction (GF%)

As per methodology, the GF% of the formulated hydrogel membranes was determined in water, as shown in Table 4. A decrease in GF% was noted with increasing concentrations of SA, PVA, and F-127. In case of SA, this effect can be the result of an increase in hydrophilic chains -COOH and -OH, which, upon interaction with water, weaken the molecular structure [54], while for PVA, this effect can be the result of a decreased PVA entanglement reaction, which in turn slows the gelation process. As PVA concentration is increased, entanglement also declines, resulting in a lower gel fraction [55]. Furthermore, formulation F2 (optimal composition from group 1) exhibited a GF of 66.67%, while formulation F9 (optimal composition from group 2) demonstrated a slightly higher GF% of 69.23%, indicating that the addition of F-127 enhances GF%. This variation can be attributed to better cross-linking or interaction among polymer components resulting from the addition of F-127, potentially enhancing drug delivery characteristics and provision of improved mechanical stability [19,56,57].

#### 3.3.4. Swelling Studies

**Figure 9:**
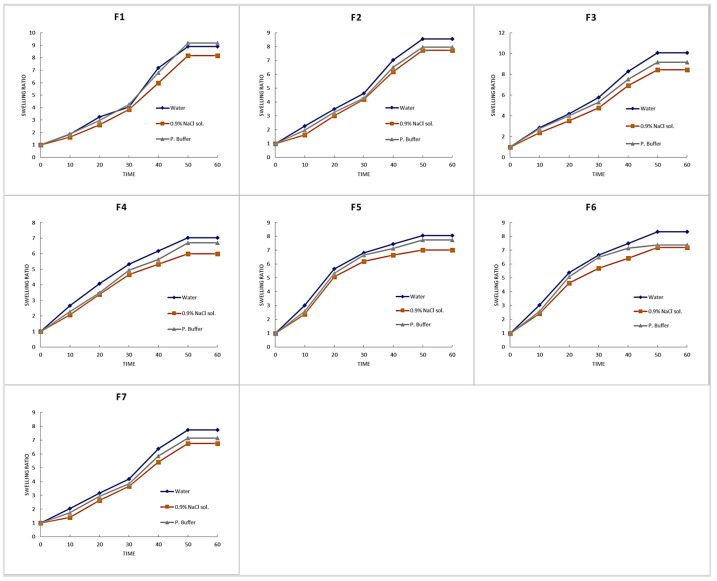
Swelling behavior of the developed hydrogel membranes in different media over time (group 1 (F1–F7)).

**Figure 10:**
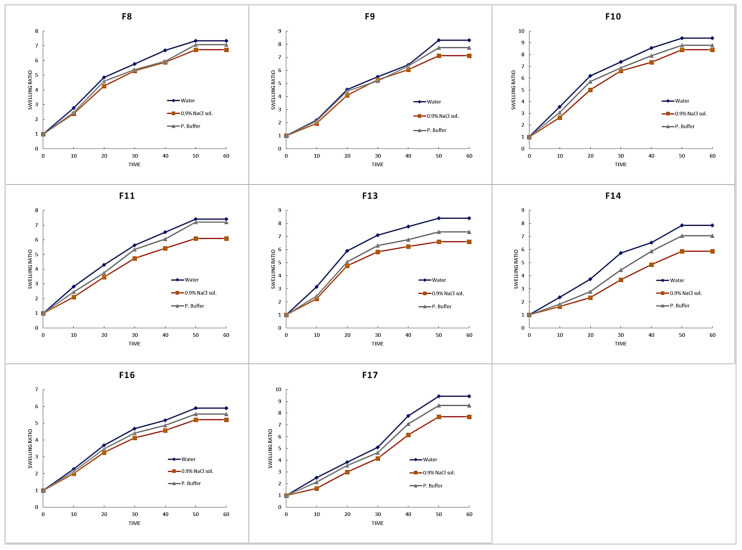
Swelling behavior of the developed hydrogel membranes in different media over time (group 2 (F8–F11, F13, F14, F16, F17)).

Different media were used for the purpose of standardization, isotonicity, and relevance to open wound pH (7.2–8.9). The results, as given in Figure 9, showed swelling tendency of the developed formulations in the order water ≥ phosphate buffer > 0.9% NaCl solution. This may be due to the hydrophilic nature of the polymers and monomers used in the development of hydrogel membranes. However, this may also be due to the presence of sodium and chloride ions in normal saline solution, which reduces the osmotic contribution to swelling pressure and the swelling rate in swelling media [57]. When the concentration of SA is raised in various media, such as water, normal saline, and phosphate buffer, the swelling ratio also rises. Figure 9 and Figure 10 show the hydrogel membrane with the maximum swelling ratio when the SA concentration is increased. These are the attributes of the hydrophilic nature of the polymer to swell in water and phosphate buffer at high rates; however, the decreased swelling ratio in normal saline is due to the presence of salts and Na+ ion exchange phenomenon [54,58].

It is added that by increasing the concentration of F-127, the formulation’s swelling decreased, as shown in Figure 10. This can be the attributes of PEO-PPO-PEO tri-block copolymers, and PPO, which forms the core of F-127 and is comparatively hydrophobic, but the poly (ethylene oxide) (PEO) side chain blocks have a hydrophilic character. PPO blocks (hydrophobic group) engaged in intermolecular links (physical cross-linking) increase with rising F-127 concentration, resulting in the formation of a transitory 3D polymer framework and a reduction in the amount of media that may enter that polymer framework, hence lowering the swelling ratio [24]. The same is the case with PVA: as the concentration of PVA is increased, the number of carboxylic groups also increases, which ultimately leads to an increase in the swelling ratio, as shown in Figure 10. F-6 and F13 had the highest concentration of PVA [25].

#### 3.3.5. In Vitro Drug Release Analysis

**Figure 11:**
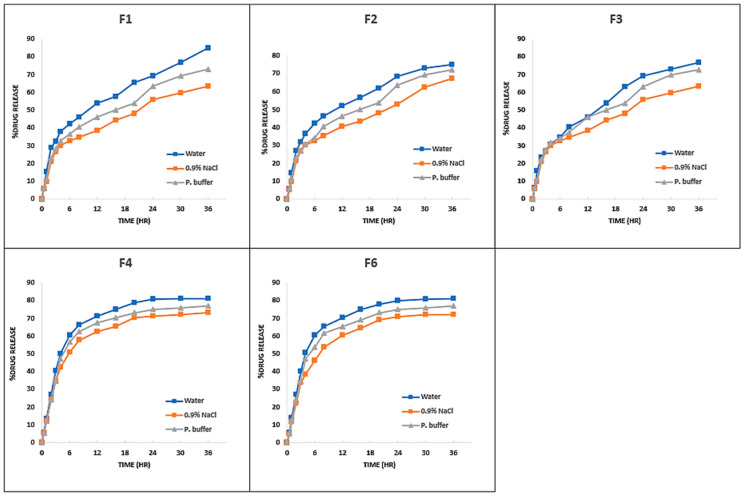
Percent drug release vs. time (h) curve of group 1 (F1–F4, F6) formulations in various media.

**Figure 12:**
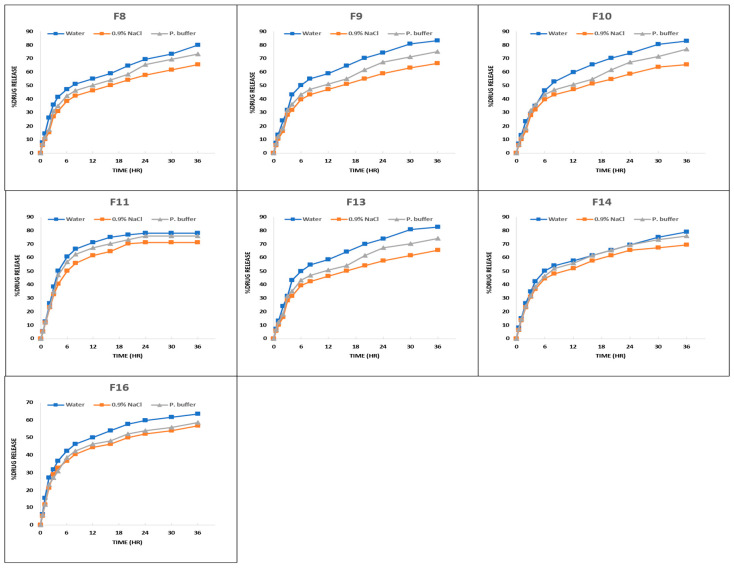
Percent drug release vs. time (h) curve of group 2 (F8–F11, F13, F14, F16) formulations in various media.

The developed drug-loaded hydrogel membranes contained variable concentrations of SA, PVA, and F-127 which effected the in vitro drug release in different media. Table 5 shows Test Statistics (K-W ANOVA) for % drug release among the developed hydrogel membranes, at a level of significance equal to 0.05. The results support the argument that the developed hydrogel membranes released the % amount of colistin in different media in a descending order as water > phosphate buffer > normal saline as shoen in. In group 1 formulations Figure 11 the percent drug release in water, normal saline, and phosphate buffer ranges at 75–85%, 63.46–73.08% and 72.12–76.92%, respectively, while in group 2 formulations Figure 12, the range was noted as 63.46–83.08%, 56.73–71.15%, and 58.65–76.92% for release in water, normal saline, and phosphate buffer, respectively. The highest drug release in water was 85% and 84% noted for F1 in group 1 and F9 in group 2. However, no significant difference was observed (*p*-value = 0.825) between the groups. The highest drug release in normal saline was 73% and 71.15% noted for F4 in group 1, and F14 in group 2, respectively. However, no significant difference was observed (*p*-value = 0.467) between the groups. The highest drug release in phosphate buffer was 76.92% and 76.92% noted for F4 in group 1 and F10 in group 2, respectively, but no significant difference was observed (*p*-value = 0.597) between the groups.

A decreasing trend in drug release was seen when F-127 concentration increased from 2% to 4%. This may be because the hydrogel composites’ diffusion coefficient drops as the concentration of Pluronic F127 rises. Previous research has shown that micelle production causes the super-molecular structure of F127 to become densely packed and diminishes the diffusion coefficient inside hydrogel composites. The observed trend might possibly be the outcome of hydrophobic methyl group exposure. The hydrophilic side chains (PEO) having -OH group that have been separated from the core hydrophobic (PPO) moiety containing methyl group -CH become exposed as the concentration of F-127 rises. The hydrophilicity is reduced through the addition of more methyl groups, which also reduces swelling and, in turn, the release of drugs from the hydrogel composites [59].

#### 3.3.6. Determination of Drug Loading

The drug (colistin) was added during the synthesis phase of the hydrogel, which allows for a uniform distribution throughout the formulations. This approach has been shown to maintain high encapsulation efficiency, as evidenced by studies reporting encapsulation efficiencies of up to 98.65% in similar formulations [5,59]. Furthermore, it is noted from the drug release studies that a significant portion of colistin is released within 24 h. This release profile does not imply loss during synthesis; rather, it reflects the intended release mechanism designed for therapeutic efficacy [5]. The sustained release observed in the formulated hydrogel membranes is indicative of successful drug loading and retention within the hydrogel structure.

#### 3.3.7. Antibacterial Studies

**Figure 13:**
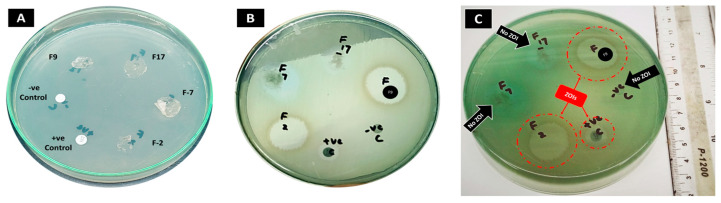
Image results: (**A**) culture plate before incubation; (**B**) culture plate after 24 h of incubation at 37 °C; (**C**) plate labeled properly showing ZOIs in F2, F9, and +ve control, while no ZOIs in F7, F17, and −ve control; ZOI: zone of inhibition.

Antibacterial studies of the developed hydrogel membranes were performed through disk diffusion method (Kirby-Bauer) against MDR *P. aeruginosa* strains, the results are shown in Figure 13 and Table 6 which shows clear ZOIs for colistin loaded hydrogel membranes F2, F9 and the positive control (colistin commercial disk); however, no ZOIs were observed for the developed blank hydrogel membranes F7, F17 and the negative control (blank filter paper disk) [5]. ZOI of F9 was superior to F2’s ZOI, and significantly higher than the positive control, indicating that colistin was intact inside the developed hydrogel membranes, and the presence of F127 potentially enhanced the antibacterial properties of F9 by improving their drug release profile. The variation in ZOIs between positive control and drug-loaded hydrogel membranes’ disks may be due to drug content variation, i.e., 10 µg and 65 µg, and difference in diameters, i.e., 5 mm and 8 mm for the positive control and hydrogel membranes, respectively [5]. This variation in ZOIs was not proportionate to the variation in drug content and size of the disks, but instead, ZOIs observed for the positive control were almost half of the drug-loaded hydrogels’ disks. Perhaps the reason was the sustained drug release pattern of the hydrogels compared to the antibiotic disks.

Colistin was successfully liberated from the polymeric matrix and remained pharmacologically active after being incorporated into both hydrogel systems, as demonstrated by the reported antibacterial efficacy against MDR *P. aeruginosa*.

## 4. Conclusions

Two groups of alginate-based hydrogel membranes loaded with colistin were successfully developed from Na-alginate, Pluronic F-127, and polyvinyl alcohol aimed at treating MDR burn wound infections. Group 1 (SA-PVA-C) comprised conventional alginate-based membranes, while group 2 (SA-PVA-F127-C) incorporated with thermoresponsive Pluronic F-127, a modern hydrogel. The developed membranes were characterized through various techniques. FTIR spectroscopy confirmed the successful incorporation of colistin without chemical interactions, ensuring its bioavailability. It was confirmed through TGA that the presence of Pluronic F-127 potentially improved the mechanical performance of the group 2 membranes. Similarly, through mechanical strength analysis, it was observed that the group 2 formulations have superior stiffness and mechanical stability, attributed to enhanced cross-linking from F-127. SEM revealed that the group 2 membranes have advantageous porosity for oxygen supply, promoting wound healing. Moreover, in vitro studies including WVTR measurements indicated that both groups maintained optimal moisture levels essential for healing. However, GF% and antibacterial efficacy studies concluded that group 2 formulations were superior with respect to drug delivery, mechanical stability, and superior antibacterial properties. Overall, this study supports the hypothesis that alginate-based modern hydrogel membranes (group 2) are more effective colistin delivery systems than group 1 for MDR burn wound applications. Further work should evaluate activity against additional wound-associated pathogens and include in vivo studies to validate performance in biological environments and explore clinical applicability.

## Figures and Tables

**Table 1:** Content ratio and number of formulations developed.

Formulation	SA (%*w*/*w*)	PVA (%*w*/*w*)	F-127 (%*w*/*w*)	Glycerol (%*w*/*w*)	Colistin (%*w*/*w*)
F1	2	5	0	7	0.025
F2	3	5	0	7	0.025
F3	4	5	0	7	0.025
F4	3	4	0	7	0.025
F5	3	5	0	7	0.025
F6	3	6	0	7	0.025
F7-blank	3	5	0	7	0
F8	2	5	3	7	0.025
F9	3	5	3	7	0.025
F10	4	5	3	7	0.025
F11	3	4	3	7	0.025
F12	3	5	3	7	0.025
F13	3	6	3	7	0.025
F14	3	5	2	7	0.025
F15	3	5	3	7	0.025
F16	3	5	4	7	0.025
F17-blank	3	5	3	7	0

**Table 2:** Details about mechanical properties of the developed hydrogel membranes F2, F7, F9, and F17 as determined by UTM analysis.

Formulation	% Elongation ± SD	Tensile Strength (MPa) ± SD	Peak Load (N) ± SD	Young’s Modulus (MPa) ± SD
F2	56.61 ± 0.91	2.43 ± 0.24	7.30 ± 0.10	1.39 ± 0.17
F7	33.90 ± 0.57	1.53 ± 0.11	4.79 ± 0.10	2.13 ± 0.10
F9	23.08 ± 0.40	1.05 ± 0.12	3.17 ± 0.10	5.71 ± 0.05
F17	27.03 ± 0.36	0.57 ± 0.03	1.72 ± 0.21	1.50 ± 0.18

**Table 3:** Thickness and solvent loss for the developed hydrogel membranes.

Formulation	Avg. Thickness (mm) ± SD	Solvent Loss (g) ± SD	Percent (%) Solvent Loss
F1	0.13 ± 0.02	28.39 ± 0.11	94.63
F2	0.14 ± 0.01	29.00 ± 0.21	96.67
F3	0.15 ± 0.02	28.50 ± 0.31	95.00
F4	0.14 ± 0.03	28.49 ± 0.24	94.97
F6	0.14 ± 0.03	28.40 ± 0.21	94.67
F7	0.12 ± 0.03	29.07 ± 0.04	96.90
F8	0.16 ± 0.02	28.58 ± 0.24	95.27
F9	0.17 ± 0.03	29.00 ± 0.21	96.67
F10	0.15 ± 0.03	28.40 ± 0.12	94.67
F11	0.15 ± 0.02	28.58 ± 0.04	95.27
F13	0.14 ± 0.03	28.60 ± 0.21	95.33
F14	0.14 ± 0.03	28.60 ± 0.22	95.33
F16	0.14 ± 0.03	28.65 ± 0.27	95.50
F17	0.13 ± 0.01	28.99 ± 0.21	96.63

**Table 4:** GF% for the developed hydrogel membranes, *W*0: initial dry weight of the hydrogel membrane before swelling; *We*: final dry weight of the hydrogel membrane after swelling and re-drying.

Formulation	*W*0 (g)	*We* (g)	*We*/*W*0	[(*We*/*W*0) × 100] ± SD
F1	0.148	0.099	0.669	66.89 ± 1.24
F2	0.138	0.092	0.667	66.67 ± 0.65
F3	0.163	0.100	0.614	61.39 ± 3.25
F4	0.158	0.108	0.684	68.35 ± 2.65
F6	0.167	0.110	0.659	65.87 ± 2.65
F7	0.131	0.086	0.658	65.80 ± 6.06
F8	0.114	0.082	0.719	71.93 ± 2.65
F9	0.130	0.090	0.692	69.23 ± 6.06
F10	0.198	0.131	0.662	66.16 ± 2.89
F11	0.182	0.130	0.714	71.43 ± 2.65
F13	0.175	0.118	0.674	67.43 ± 3.11
F14	0.120	0.094	0.783	78.33 ± 2.65
F16	0.146	0.101	0.692	69.18 ± 2.89
F17	0.147	0.101	0.685	68.50 ± 3.11

**Table 5:** Test Statistics (K-W ANOVA), for % drug release among the developed hydrogel membranes, at level of significance (0.05).

S. No.	Media Used for Drug Release Studies	*p*-Value (for F1–F16)
1.	Water	0.820
2.	Normal saline	0.467
3.	Phosphate buffer (pH 7.4)	0.597

**Table 6:** Descriptive Statistics of antimicrobial response of the developed hydrogel membranes against *P. aeruginosa*. D: diameter; f: frequency; Avg = average/mean; SD: standard deviation; Min.: minimum; Med.: median; Max.: maximum; ZOI: zone of inhibition.

Formulation	D (mm)	f.	Avg. ZOI (mm)	SD	Min. ZOI (mm)	Med. ZOI (mm)	Max. ZOI (mm)
F2	8	3	20.33	2.51	18	20	23
F7	8	3	0	0	0	0	0
F9	8	3	21.66	2.08	20	21	24
F17	8	3	0	0	0	0	0
+ve control	5	3	12.66	0.57	12	13	13
−ve control	5	3	0	0	0	0	0

## Data Availability

The original contributions presented in this study are included in the article. Further inquiries can be directed to the corresponding author.
